# Does the World Need Plant Medicines?

**DOI:** 10.3390/medicines5020039

**Published:** 2018-04-23

**Authors:** James David Adams

**Affiliations:** School of Pharmacy, University of Southern California, Los Angeles, CA 90089-9121, USA; jadams@usc.edu

Our species has always used plants as medicines, during our 200,000 year existence. This is made obvious by the plant medicines found in mummy burials, around the world, dating back thousands of years. Human beings evolved using plant medicines to stay alive. This has resulted in a genetic selection in which those who were healed by plant medicines and survived were able to pass on their genes. Modern humans must continue to use plant medicines.

When a Traditional Healer is asked how her people learned how to use a specific plant medicine, the answer is usually that God taught them. Each of us has a spiritual sense that can be used to seek answers [1]. Finding a new plant medicine involves fasting, praying, and spending as much as four days with the plant that will become a new medicine [2]. Traditional Healers are still the major or only source of healthcare for many regions of the world. The loss of Traditional Healers in some areas has resulted in major changes in healthcare, sometimes to the detriment of the people [3,4]. Since Traditional Healers may be religious leaders also, they provide moral direction for the community. The displacement of traditional religious beliefs has been detrimental in some regions.

The most powerful medicine each person has is the human body. The body heals itself. When the body is in balance, it can heal itself of many conditions, in other words, it can prevent many diseases [5,6]. Patients should be taught what balance is and how to maintain balance, including staying thin and strong.

Drugs are not magic bullets that cure diseases. They help the body heal itself. Even antibiotics and anticancer drugs are of no use when the immune and defense systems are not functioning well. In many clinical trials, the placebo works as well as the drug because of the body’s ability to heal itself [7].

Clinical trials are useful to demonstrate what a plant medicine can be used to treat and to demonstrate safety. Useful information can be derived from placebo controlled clinical trials, from clinical trials that compare a new medicine to an old medicine and from other clinical trial designs. Many different clinical trial designs are appropriate. The double blind, randomized, placebo controlled clinical trial should not be used as the gold standard of clinical trial design [7]. Many other designs are just as valid and may be less risky to patients, such as patients who are put on the placebo.

Clinical trials are frequently abused. Drugs are shown to help with one disease symptom and are approved for use, even though they are very toxic. Many of the oral medicines used to treat pain are dangerous drugs that kill many patients. Oral opioids kill about 67,000 people in the USA every year. Oral nonsteroidal anti-inflammatory drugs have been estimated to kill at least 50,000 USA patients every year from ulcers, heart attacks, and strokes [8,9]. The most dangerous way to treat pain is with oral or injected pain medications. Some of the drugs approved for type 2 diabetes cause heart disease. Some of the drugs approved for heart disease cause type 2 diabetes. Just because a drug is useful for one symptom, does not mean the drug should be used.

Drugs are frequently used as if they were the only possible treatment for a disease. Patients seem to have become convinced that drugs can take care of any disease. Prevention is the best treatment for many chronic diseases that plague the modern world. Type 2 diabetes, heart disease, and osteoarthritis are caused by excess visceral fat, obesity [10,11]. Visceral fat secretes toxic lipids and akipokines that cause these diseases. Toxic adipokines also potentiate the formation of cancer in the body [10]. As modern man has become more sedentary and obese, these diseases have increased greatly, much to the benefit of the pharmaceutical industry. A better option is to live in balance and prevent diseases.

In the midst of our crisis of drug over use and abuse, patients must find alternative choices for healthcare. Prevention should always be the healthcare of choice. When medicines are needed, many plant medicines continue to be available in most of the world. In the United States, medicines are tightly regulated by the Food and Drug Administration. Due to this strict regulation, few indigenous American plant medicines are readily available. Patients must find Traditional Healers who still know how to make Native American plant medicines [2]. Plant medicines made from European plants are much more available in the United States.

In the current publication, Edenta, Okoduwa, and Okpe teach about the use of plant medicines in Nigeria [12]. *Musa acuminata* is a species of banana that originally comes from Southeast Asia and has been cultivated for over 8000 years. It was probably introduced into Nigeria long ago. Traditional Healers in Nigeria use *M. acuminata* peels in the treatment of their patients. There are several cultivars of *M. acuminata*, each with different pharmacology and toxicology. The work of Edenta and coworkers demonstrates that some plant medicines may not be safe for human use.

Kinda et al. discuss the use of plant medicines to treat neuropsychiatric conditions by Traditional Healers in Burkina Faso [13]. Neuropsychiatric conditions existed during ancient times and were a burden for the community. In traditional villages, each member of the village has a job that is critical to the survival of the village. If a neuropsychiatric condition prevented a village member from being productive, the Traditional Healer had to find a way to help. In Burkina Faso, Traditional Healers use 66 different plant species to make medicines for neuropsychiatric conditions. Modern medicine relies on the use of dopamine 2 receptor antagonists to treat psychosis. These drugs cause extrapyramidal side effects in 50% of patients, weight gain in most patients, and potentially irreversible tardive dyskinesia in 68% of patients treated for 25 years. Even though dopamine 2 receptor antagonists are useful in neuropsychiatric conditions, their toxicity is a concern. Modern medicine may be wise to learn from the Traditional Healers of Burkina Faso.

Tchuenmogne et al. describe their work to find biologically-active compounds from *Terminalia mantaly*, the Madagascar almond [14]. They describe several compounds that are interesting antifungal agents. Pathak, Upreti, and Dikshit provide evidence of antifungal compounds from a lichen, *Usnea orientalis* [15]. The treatment of fungal infections has become very important and difficult due to the huge increase in type 2 diabetes in the world. Diabetics are very susceptible to fungal infections.

Balogun et al. examine an endangered medicinal plant, *Oncoba spinosa*. This small tree grows in many areas of Africa [16]. The fruit is used in Traditional Healing in Nigeria. Although several reports have examined the Phytochemistry of the plant, Balogun and coworkers found a compound not previously seen in the leaves. The compound, flacourtin, is also found in an Indian plant that is used in Traditional Healing.

Al-Tamimi, Rastall, and Abu-Reidah report on the antioxidant and toxic properties of nine essential oils [17]. In order to produce an essential oil, a large amount of plant material is heated, such as in a steam bath, until the volatile compounds from the plant distill. Essential oils have been used in European Traditional Healing. Some essential oils are very toxic, even to the skin. El-Tamimi and coworkers found that ginger, chamomile, and African rue essential oils were of interest. They also found that some essential oils are not pharmacologically potent and suggest that storage and preparation conditions may be important with these products.

Ouedraogo and Kiendregeogo discuss the antibiotic potency of *Anogeissus leiocarpus*, African birch [18]. The plant is used by Traditional Healers in wound healing. Methanol extracts of the plant were examined against bacterial strains. This work shows the importance of wound healing in traditional villages. There are probably several alternative treatments that modern medicine should consider for the treatment of difficult wounds, such as diabetic skin ulcers.

Santos et al. present evidence of cytotoxic and antimicrobial compounds in an essential oil from *Lippia alba*, bushy mattgrass, which comes from the Caribbean, Central and South America [19]. They found 39 compounds, of which many are monoterpenes. Some monoterpenes from bushy mattgrass may be useful against fungal infections.

Khurm et al. provide evidence of the safety of *Heliotropium strigosum*, called Kharsan, Gorakh pam, and Bhangra in Pakistan [20]. The plant is used in Traditional healing for respiratory and GI problems. Plant preparations have low toxicity against several bacteria and fungi. The medicine is known to contain compounds that relax inflamed lung tissue.

Work from my laboratory, by Wang et al., provides controversial evidence for the use of a plant medicine in the treatment of Alzheimer’s disease [21]. In ancient times, some older people suffered from what is now called Alzheimer’s disease. Chumash Healers found that the berries of *Heteromeles arbutifolia*, toyon, a California plant, helped slow down the progression of the disease. White people labeled the plant poisonous, due to their racist beliefs that plants used by Indians must be poisonous. The plant has not been carefully examined until now. My group found several interesting compounds in the plant and demonstrated the safety of the berries in several patients. All of the clinical trials of new Alzheimer’s disease drugs have failed [22]. Perhaps this plant medicine should be examined in clinical trials.

Olorunnisola, Fadahunsi, and Adegbola teach us about Traditional Healing with *Sphenocentrum jollyanum*, aduro kokoo. The plant is used as a medicine in several West African countries [23]. Alkaloids, saponins, flavonoids, and other compounds are found in the plant. Traditional Healers have found several uses for the plant in treating their patients, including high blood pressure. Black Americans are resistant to several antihypertensive drugs. It may be wise to use this plant in resistant patients.

Rodriguez Villanueva, Esteban, and Rodriguez Villanueva discuss the use of *Marrubium vulgare*, horehound in type 2 diabetes [24]. The authors find that clinical trials performed with the plant were flawed. They suggest finding better clinical trial methods in order to accurately assess the value of the plant. This is a constant problem in plant medicine clinical trials. In the USA, the Food and Drug Administration and the National Center for Complementary and Integrative Health insist on using hydro-alcoholic extracts of American plants or similar preparations in clinical trials, even though Traditional Healers have never used these preparations. The purpose of these clinical trials, therefore, is to disprove the efficacy of the plant medicines. This is a vivid reminder of the anti-Indian racism that permeates these federal agencies.

Teng et al. provide a case report of a woman who had a successful pregnancy after being treated with Chinese herbal medicines. In vitro fertilization had failed in the woman [25]. This case reinforces the power of herbal medicines.

The purpose of the current writing is to educate the healthcare community about the importance of plant medicines. Plant medicines can be safe and efficacious even when modern drugs fail. Plant medicines should be used in the traditional preparations and by the traditional routes. One of the advantages of plant medicines is that they contain several active compounds that all add to therapy. They may even potentiate or synergize the effects of other compounds in the medicine. The author of the current writing encourage the modern use of plant medicines after performing realistic clinical trials.
